# The Pandemic, Patient Advocacy, and the Importance of Thinking Comment on "The Rise of the Consucrat"

**DOI:** 10.34172/ijhpm.2020.114

**Published:** 2020-07-08

**Authors:** Órla O’Donovan

**Affiliations:** School of Applied Social Studies, University College Cork, Cork, Ireland.

**Keywords:** Patient advocacy, Pandemic, Necropolitics, Catastrophe Capitalism, Consucrats, Cosmedics

## Abstract

The profound inadequacies of Western modernist ways of thinking have been revealed by the intimately connected catastrophes of climate destruction, and more recently, the coronavirus crisis. The pandemic has forced us to notice deepening inequalities and has generated troubling questions about its causes, and who and what can be sacrificed in a pandemic. The analysis offered in Evelyn de Leeuw’s essay "The rise of the consucrat" suggests that the particular type of patient advocates she calls consucrats are unlikely to engage in thinking together about these urgent questions. If anything, due to their narrow biomedical focus and alliances with the pharmaceutical industry, they are likely to facilitate catastrophe capitalism. However, within the field of patient advocacy, there is a diversity of ways of thinking, occasionally leading to bitter contention. A number of terms is needed to reflect this diversity. One group of patient advocates who have come to the fore in recent times might be called medical cosmopolitans, or cosmedics, those who are challenging opportunistic catastrophe capitalism during the pandemic and advocating for global access to essential medicines. Forcing us to notice our deep interdependencies and entanglements, the pandemic has revealed how ludicrous it is to think about patients as consumers, and the need to think about and imagine more-than-human patient advocacy.

## Introduction - the importance of thinking in the Capitalocene

As part of the exhibition and conference Critical Zones – Observatories for Earthly Politics that took place in May 2020, the feminist philosopher Donna Haraway was interviewed about the ideas in her many books, including *Staying with the Trouble: Making Kin in the Chthulucene*.^1^ Central to Haraway’s work is the assertion of the importance of finding new ways to think, given the profound inadequacies of conventional ways of thinking revealed by what she refers to as the slow catastrophe of climate destruction. For her, there is an urgent need to refuse to reproduce the Western modernist thinking that led to the Capitalocene; this is the term she uses to refer to our current era of life-destroying capitalism and its imperatives of infinite extraction and growth. The obligation to find better ways of thinking, and living and dying, has been further underlined by what she refers to as the fast catastrophe of the coronavirus disease 2019 (COVID-19) pandemic. These fast and slow catastrophes are intimately connected. In the United States, where she lives, the most extreme scene of destruction from the pandemic is among the Navaho nation. Arguing that we need to take seriously connections and entanglements over time and space, this destruction is in no small part a consequence of the ongoing history of colonial capitalist extraction of water, coal and uranium from the lands of the Navaho. The pandemic has taken place during a right wing turn and conservative revolution globally, featuring what she describes as a “necrophilic disregard for the lives of those who have no value.” At a time of intensifying mass immiseration of humans and mass extinction of non-humans, how to live less destructively is an urgent question that requires thought.

It was just over a fortnight after the World Health Organization had declared the COVID-19 pandemic when I received an invitation from the editor of the *International Journal of Health Policy and Management* to write a commentary on Evelyne de Leeuw’s essay “The rise of the consucrat.”^2^ She proposes the concept of *consucrats* to refer to healthcare “career consumer representatives.” The word fuses parts of the neoliberal designation of the patient as a healthcare “consumer” and the pejorative term “femocrat,” or the even more disparaging and racist appellation “abocrat,” used to refer to representatives of women and Australian Aboriginal people in state bureaucracies. De Leeuw offers consucrat as a term to refer to “a new class” of “Consumer health advocates [who have] become complicit in extenuating the perverse effects of the medical-industrial complex,” a complex that has acquired enormous economic and cultural power in the Capitalocene. At the time I received the invitation, fear and concerns about the fast coronavirus catastrophe were rife internationally. In Italy deaths from the feral virus had already exceeded 10000, and images of hospital intensive care units, overcrowded with seriously ill patients on ventilators, had become a media staple. Distressing necropolitical questions had come to the fore, evident in headlines such as “Spanish doctors are forced to choose who to let die” (see https://www.bloomberg.com/news/articles/2020-03-25/spanish-doctors-forced-to-choose-who-to-let-die-from-coronavirus). In Ireland, where I live, unprecedented authoritarian public health restrictions had been introduced, along with a new household lexicon. I was staying with my mother and father, who has advanced stage Parkinson’s disease, to help them “cocoon” (“ag neadú” or nesting in Irish), in line with government appeals to people aged over 70 not to leave their homes. It was not long before the necropolitics of the response in Ireland to the crisis became apparent, resulting in calls for a public enquiry into why so many deaths from COVID-19 occurred in private nursing homes for older people. Similar to the Navaho territories, the pandemic forced us to notice that many nursing homes are “sacrifice zones,” segregated spaces for people whose lives do not matter.^3^ Alongside the many celebrations of the outbreaks of neighbourly care prompted by the pandemic, the proliferation of critical commentaries about the unleashing of authoritarianism precipitated by the pandemic had begun, together with troubling questions about its causes, and who and what can be sacrificed in a pandemic. De Leeuw’s essay suggests that the particular type of patient advocates she calls consucrats are unlikely to think together about these urgent questions.

## Troubling the Rise of Patient Advocates and Organisations

The rise of increasingly well-financed, professionalised and globalised patients’ organisations is both welcome and troubling and has generated the need for new concepts to help us think about and understand its consequences. The rise of patients’ organisations funded by and aligned to the pharmaceutical and medical devices industries is particularly troubling during a pandemic which has created new opportunities for catastrophe capitalism. As pointed out by Evelyn de Leeuw, much patient advocacy is disease-specific and has an exceedingly biomedical focus, centred on demands for the speedy development and access to new diagnostic tests and treatments. Her concern is that this narrow focus stands in the way of efforts to address the “‘upstream,’ ‘distal,’ political, social and commercial causes of ill health in populations.” Put differently and drawing on Donna Haraway’s ideas sketched out above, the narrow focus of many patients’ organisations can work against thinking about the entanglements across time and space that produce diseases and the consequences and inequalities connected to producing and consuming more medicines. Hers is a call to reassess thinking as usual, which in the case of many of the advocates and organisations that are the focus of de Leeuw’s analysis is that they are involved in a *war* on disease, and the corporations of the medical-industrial complex are their allies. (This is also the prevailing way of thinking about the coronavirus crisis: the biotech and pharmaceutical industries are thought to be the source of medical munitions in the war against the virus, where the pandemic is understood as “an arms race between species”).^4^ This is not to deny the value and significance of disease-specific advocacy and organisations. Mutual support and knowledge sharing and production by people with a common diagnosis is a hugely important aspect of many patients’ organisations, as attested by my parents’ experience of local support groups affiliated to the Parkinson’s Association of Ireland. Patient representatives and their organisations have been crucial to revealing the lethal consequences of the privatization of medicine, such as the outsourcing of cytology testing to US corporations by the Irish national screening programme CervicalCheck.

 It is important to acknowledge the historical roots of much contemporary patient advocacy in social movements, such as the women’s movement and the disability movement, that challenged conventional understandings of health and produced alternative ways to think about the benefits and harms of medical interventions.

Emphasising that much remains unclear about patients’ experiential knowledge and the credibility and authority it should be accorded, Stuart Blume has considered its emergence as a form of cultural capital used to legitimate demands for greater inclusion of patients in healthcare decision-making. Even though the rhetoric of listening to patients is pervasive, he argues their “experience is treated as authoritative, as worthy of being characterized as ‘knowledge’ only to the extent that it appears compatible with medical knowledge and assumptions.”^5^ When patients and their organisations depart from conventional ways of thinking, their status in the medical-industrial complex tends to be significantly diminished. A recently published account by Sharon Batt^6^ illustrates how when patient advocates’ think seriously about conflicts of interest in pharmaceutical industry sponsorship, they can meet hostility from other patient advocates. Author of the book *Health Advocacy Inc.*, Batt describes her experience of being effectively censored at a Canadian national health technology assessment conference. The paper she was prevented from delivering at the conference, due to aggressive heckling from patient advocates, was prompted by her observation that at other similar conferences most patients were canvassing for public subsidization and access to new drugs. Furthermore, she notes “I had rarely, if ever, heard patients speak … about drug safety and efficacy, about the impact of rising drug prices on the viability of the public healthcare system, or about systemic biases in clinical trials.” She attributes the unquestioning homogeneity of these patient advocates’ contributions to policy discussions to the significant sponsorship they receive from pharmaceutical corporations, noting that patient organisations willing to question and critique the industry struggle to survive. In keeping with this analysis, Evelyn asserts many consucrats have become “co-opted apparatchiks,” with questionable representational legitimacy. However, Sharon Batt’s analysis points to the need for multiple concepts to think about the diversity, messiness and bitter contention in the field of patient advocacy, as she herself is a longstanding breast cancer activist and founding member of Breast Cancer Action Quebec.

## AIDS and COVID-19 Technology Pool Advocates as Cosmedics

For many decades, US AIDS treatment activists in the 1980s and the organisation ACT UP, the AIDS Coalition to Unleash Power, have served as a model for health advocates mobilised around many other conditions.^7^ These were disease-specific activists who campaigned for access to treatments and represented patients in statutory bureaucratic forums, but the descriptor of consucrats strikes me as inappropriate. Their cause extended in many directions, including challenging high prices of medical treatments, orthodoxies in how clinical trials were conducted, and the stigmatisation of people with HIV/AIDS. Additionally, they opposed and exposed the catastrophic consequences of reliance on the market in the HIV/AIDS epidemic. As pointed out by advocates of a COVID-19 technology pool,^8^ in 1996, antiretroviral medicines that transformed HIV/AIDS from being a death sentence to a chronic condition, were launched on the US market by a number of pharmaceutical companies. However, because of patent-protected high prices, for many sufferers internationally, it was the equivalent of there being no treatment. It was not until eight years later, during which time millions had died from the virus, that sufferers in India and South Africa could afford the drugs. Without a COVID-19 pool that would encourage governments to share medical innovations and make them available globally, without the restrictions of borders or patents, they argue history will repeat itself and “Pharmaceutical giants will bury treatments in a thicket of patents, making them unaffordable to the world’s poorest.” Rather than being consucrats, these opponents of catastrophe capitalism and advocates for global access to essential medicines might be called medical cosmopolitans, or *cosmedics*.

## Interdependent Patients, not Independent Consumers

 De Leeuw’s paper usefully highlights a troubling trend in patient advocacy, but the term consucrat is also troubling. Surely the fast catastrophe of the pandemic has shown us once and for all that use of the term consumer to refer to patients is wrong? It is preposterous to imagine the many people seriously ill with the virus, such as those on ventilators in intensive care units or those sent directly for palliative care, or worse still, those who receive no medical care, as independent market-minded shoppers, calculating the costs and benefits of various healthcare choices on offer. The global health crisis has punctured the illusion of the autonomous individual, that mythical figure in modern Western understandings of what it is to be human. Interdependence is the order of the day, as we have become acutely aware of our reliance on others to minimise our risk of infection with the virus, and to care for us if we do. Although Evelyn de Leeuw uses the language of the healthcare consumer, and incorporates some of it in the word consucrat, she accepts it is not without its problems. Amongst these is that it oversimplifies “the realities of what makes and maintains health, and creates or sustains disease. This worldview rhetorically dismisses other players in the medical-industrial complex such as finance and insurance companies, Big Pharma and Big Tech, and a critical role for government.” What the pandemic has shown us is that the assemblage of players that shape our experiences of health and disease extends beyond humans. Recognition of these entanglements is evident in the Trump Administration’s Coronavirus Task Force member, Anthony Fauci’s statement that “Ultimately, the virus will really determine when we can safely reopen.” Not only has the pandemic ruptured modernist illusions of “Man” having mastered nature, it has also highlighted the importance of a more-than-human understanding of the determinants of health, and approach to health and patient advocacy. There is much to think about.

## Ethical issues

 Not applicable.

## Competing interests

 Author declares that she has no competing interests.

## Author’s contribution

 OO is the single author of the paper.
